# Conducting materials as building blocks for electronic textiles

**DOI:** 10.1557/s43577-021-00117-0

**Published:** 2021-06-17

**Authors:** Anja Lund, Yunyun Wu, Benji Fenech-Salerno, Felice Torrisi, Tricia Breen Carmichael, Christian Müller

**Affiliations:** 1 Department of Chemistry and Chemical Engineering, Chalmers University of Technology, Gothenburg, Sweden; 2 Department of Chemistry and Biochemistry, University of Windsor, Windsor, Canada; 3 Molecular Sciences Research Hub, Imperial College London, White City Campus, London, UK; 4 Wallenberg Wood Science Center, Chalmers University of Technology, Gothenburg, Sweden

**Keywords:** Fabric, Metal, 2D materials, Polymer, Sustainability

## Abstract

**Abstract:**

To realize the full gamut of functions that are envisaged for electronic textiles (e-textiles) a range of semiconducting, conducting and electrochemically active materials are needed. This article will discuss how metals, conducting polymers, carbon nanotubes, and two-dimensional (2D) materials, including graphene and MXenes, can be used in concert to create e-textile materials, from fibers and yarns to patterned fabrics. Many of the most promising architectures utilize several classes of materials (e.g., elastic fibers composed of a conducting material and a stretchable polymer, or textile devices constructed with conducting polymers or 2D materials and metal electrodes). While an increasing number of materials and devices display a promising degree of wash and wear resistance, sustainability aspects of e-textiles will require greater attention.

**Graphical abstract:**

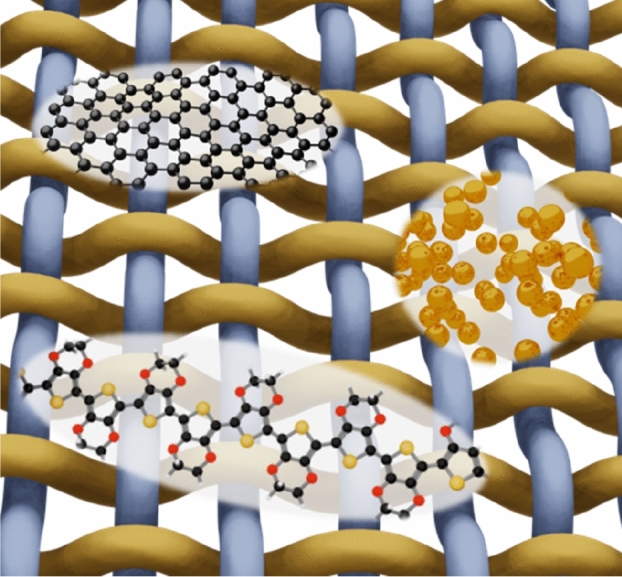

## Introduction

Electronic textiles (e-textiles) represent a fascinating technology platform as they may facilitate futuristic applications which not long ago only existed in science fiction movies. Increasingly, advanced functionality (e.g., chemical and physical sensing) can be realized with electrically conducting fibers and fabrics.^1,2^ It seems inevitable that the Internet of Bodies (IoB), which is today limited to small wearable devices such as smart watches or hearing aids, will soon include the textiles that already surround us every day (e.g., clothing, upholstery, and medical aids). E-textiles will not only be able to register our pulse, temperature, and movements,^3,4^ but also analyze our skin and sweat,^5,6^ process the data^7,8^ and transmit them via textile antennas,^9^ or communicate via textile keyboards,^10,11^ and displays.^12,13^ Further, energy-harvesting textiles will be able to convert body heat,^14^ biomechanical movement,^15,16^ and sunlight^17^ into electricity, which can be stored in textile batteries and supercapacitors^18^ until it is needed to power the textile-based electronic devices.

**Figure 1:**
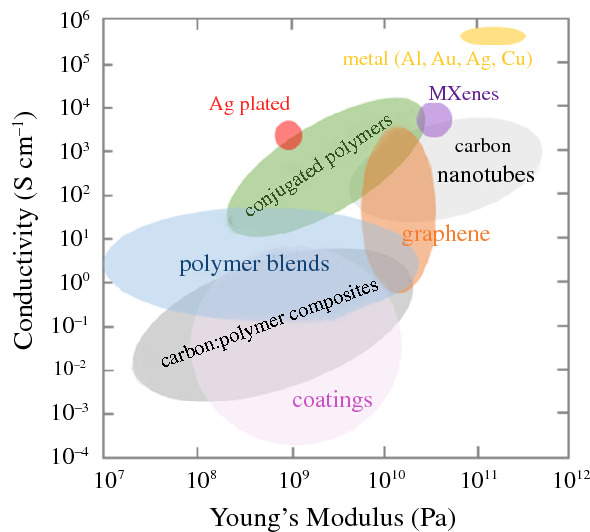
Volume electrical conductivity as a function of Young’s modulus for electrically conducting fibers or yarns based on metals (yellow) such as aluminum (Al), gold (Au), silver (Ag) and copper (Cu), MXenes (purple), carbon nanotubes (light gray), graphene (orange), composites of a carbon allotrope and a polymer (dark gray), doped conjugated polymers (green) or blends of a conducting and insulating polymer (blue), as well as insulating fibers or yarns with a conducting polymer or carbon allotrope coating (pink) and Ag plated yarns (red); the Ashby plot was constructed based on data from References 21–23.

Metals, whose electrical conductivity of about 5 × 10^5^ S cm^–1^ is unrivalled (**Figure ****1**), can be used to create decorative designs within textiles; embroidering gold fibers into textiles, also known as Zardozi, has been used for centuries. E-textiles that are already in use today comprise electroplated silver coatings or incorporate thin stainless-steel fibers, and thanks to recent advances new applications are now emerging. Other commercial e-textile solutions are based on composites of carbon black and a polymer, which can be used as a textile coating or are spun into fibers.

To realize e-textiles with the whole suite of envisaged functionalities, it will be necessary to employ a wider library of materials that not only includes electrical conductors, but also semiconductors and electrochemically active materials. There are a number of materials such as conjugated polymers, carbon allotropes, including carbon nanotubes and graphene, as well as 2D materials such as MXenes that can be directly spun into conducting or semiconducting fibers. The resulting fibers tend to display a high conductivity, but also a high Young’s modulus (Figure 1), which is not suitable for the fabrication of pliable textiles, since brittle materials would fracture during, for example, knitting. The mechanical and electrical properties can be decoupled by blending the (semi)conducting material with an insulating polymer. Nanocomposites of carbon allotropes as well as blends of conjugated and insulating polymers allow to access the full range of mechanical behavior from stretchable and elastic to ductile and tough (composites and blends in Figure 1), albeit at the expense of a high electrical conductivity, which is typically limited to about 10 S cm^−1^. Some materials such as conjugated polymers, graphene and MXenes can undergo redox reactions, which are particularly intriguing for energy-storage applications, but also bioelectronics, where mixed conductors are needed that can transport both electrons as well as ions.^19,20^

In this article, we focus on metals, conducting polymers, and 2D materials such as graphene and MXenes to highlight some of the progress that has recently been made in the context of e-textiles. Several of the most advanced devices that we will discuss combine different types of materials, which ultimately poses a challenge with regard to recycling. To ensure that e-textiles are designed with sustainability in mind it will be necessary to consider the full life cycle of this exciting new technology platform already at the early stages of development.

## E-textile building blocks: From fiber to fabric

**Figure 2:**
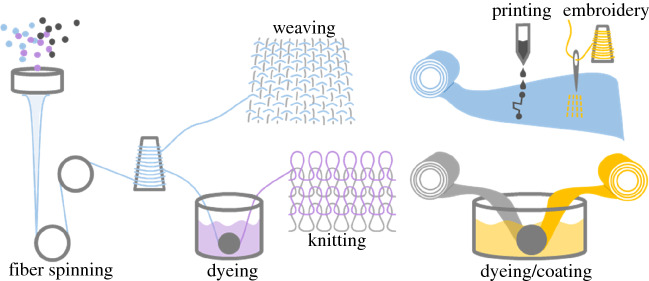
Schematic of the textile manufacture process; an insulating polymer, a conducting material, or a composite or blend of those constitutes the raw material for fiber spinning. This is followed by yarn twisting (not shown), and the yarn in turn may be dyed with a functional ink, and/or converted into a fabric by weaving, knitting, or felting (not shown). Fabrics can be further functionalized by printing, embroidery, or dyeing/coating.

A textile is defined, rather broadly, as a piece of material consisting of textile fibers. A textile fiber, in turn, is thin, flexible, and of a high aspect ratio (length over diameter). In practice, textile fibers typically have a diameter in the range of 10 µm to 50 µm, and a length of 15 mm to 150 mm. This matches the dimensions of commonly used natural fibers, including cotton and wool, for which industrial yarn spinning equipment is optimized. Synthetic fibers may be virtually endless, in which case they are called filaments, but are often cut into shorter staple fibers with the dimensions previously mentioned. Fibers are spun into yarns or threads (thinner yarns), which can subsequently be used to form fabrics by weaving, knitting, or felting processes (**Figure ****2**).

A textile is a hierarchical material, and for e-textile applications the conducting, semiconducting, or electrochemically active component can be integrated at any level. Metals can be incorporated at the fiber level by the use of thin metal wires, either in their pristine form or co-mingled with insulating textile fibers. For wearable applications, it is more common to add metals as a thin layer on the surface of a conventional textile fiber. Electrically conducting textile fibers can also be solution spun from semiconducting polymers, carbon nanotubes, and 2D materials such as graphene and MXenes. The electrical and mechanical properties of conducting fibers and yarns tend to increase in tandem (Figure 1). Consequently, fibers with a high conductivity of more than 10^3^ S cm^–1^ tend to exhibit a Young’s modulus of more than 10–100 GPa, which by far exceeds values typical for natural fibers (e.g., *E*_*wool*_ ≈ 0.2 GPa and *E*_*cotton*_ ≈ 0.6 GPa).^24^ A high stiffness may be sought after in technical textiles, but for wearable applications, it is less attractive as it makes fabrics feel abrasive, prickly, and of poor conformity. Moreover, for use in textile processing equipment for weaving, knitting, and sewing, fibers are required to be bendable as well as reversibly stretchable to a strain of at least 5 percent. The weaving and sewing processes in particular will subject textile yarns to a substantial amount of abrasion as they will be passed repeatedly through a weaving shed or through a needle’s eye.

The correlation between mechanical and electrical properties can be decoupled by combining the conductive component with an insulating material, for example, by twisting metal staple fibers with traditional textile fibers to form a yarn. Further, highly stretchable conducting fibers and yarns can be created using blends or composites with a low Young’s modulus matrix polymer such as styrenic block copolymers or a variety of polyurethanes.^25^ However, blending with an insulating material typically limits the final electrical conductivity to about 10 S cm^–1^ (Figure 1).

Once robust electronically and electrochemically active yarns are available, intricate e-textile devices can be manufactured and different types of devices can be integrated into the same fabric by combining different types of yarns using standard textile techniques. For example, embroidery can be used to create energy-harvesting textiles by stitching conducting yarns through a thick insulating fabric to form the many “legs” required for a thermoelectric device.^22^ Weaving can produce logic circuits; for example, Bae et al. combined a pristine cotton yarn with yarns coated with aluminum and polyethylene glycol dimethacrylate into a woven memory textile, where each intersection of the coated yarns formed a memristor.^7^ Knitted textiles are intrinsically stretchable, as the yarns in the fabric are connected by loops incorporating a large degree of free volume, and are uniquely suited for strain sensing devices where both, the deformation of the yarns and the modulated interconnectivity of the fabric, result in a measurable resistance change upon stretching.^25^ Intriguingly, the different intrinsic mechanical properties of woven and knitted textiles have been exploited to develop textile actuators for use as artificial muscles with modulated strain (knitted fabric) or stiffness (woven fabric).^26^

Alternatively, an existing textile cloth of natural or synthetic fibers can be dyed, coated, or printed with an electrically conducting material to form a conducting textile.^27^ By printing or coating onto a conventional yarn or fabric, e-textiles can be achieved with retained textile “feel” (i.e., the mechanical properties of the base materials are preserved). Moreover, the printing process has the advantage of offering direct patterning of e-textile devices onto the textile surface. A challenge with this textile-as-substrate approach is the three-dimensional and porous structure of textiles, which comprises an interwoven pattern of yarns interspersed with voids. The voids provide space for deformation and movement of the yarns with strain, which makes the textile flexible and stretchable, but also disrupt any added surface layer. Traditional physical vapor deposition (PVD) methods used to deposit metal films on 2D planar surfaces in microelectronics fabrication are not suitable for textiles due to the line-of-sight nature of the process.^28^ A polymeric planarization layer (e.g., composed of polyurethane) can be applied to the fabric surface to make it compatible with modern microelectronic fabrication techniques.^29,30^ For example, fabrics passivated with a planar layer have been used for the fabrication of a number of different solar cell textiles, including organic, dye sensitized, and perovskite solar cells.^17^ However, the planarization layer diminishes the intrinsic softness and stretchability of fabrics by filling the voids and restricting yarn mobility. Moreover, to achieve a robust coating it is important that the conducting material enters the voids within the textile and, ideally, binds chemically to the individual fibers.

For wearable e-textiles to be practical and useful on a large societal scale, they need to be not only comfortable, but they must also be able to sustain many cycles of wear (including abrasion and stretch) and wash. The potential release of small molecules and particles from e-textiles during domestic (water-based) laundry may not only compromise the functionality, but also cause harm to the environment. We note that several promising studies on washable e-textiles are available,^14,31–33^ but more work that follows standardized protocols is needed to develop truly robust e-textile materials.

## Metals

Metals offer a very high conductivity of about 5 × 10^5^ S cm^–1^ (Figure 1), making them useful as interconnects and electrodes due to minimization of Ohmic losses. Although metals are not biodegradable as some conducting polymers and carbon-based materials are, they are sustainable because metals are accessible and highly recyclable materials.^34^ Some metals, such as gold, are also biocompatible, which is important for wearable e-textiles worn against the skin. These properties make metals promising as conducting building blocks for e-textiles.

For the development of e-textiles, metal wires have been used as conductive building blocks within fabrics.^35^ The main issue with this approach, however, is that metal wires are usually stiffer and heavier than regular textile fibers, which can compromise the wearability. This problem can be alleviated by coating conventional insulating fibers with metal-based nanomaterials, such as thin metal films^36,37^ and metal nanowires,^38^ which retains the pliability of the insulating fiber while still maintaining metallic conductivity. Metal-based nanomaterials have also been coated on conductive fibers made from carbon materials to enhance their electrical properties.^39^ Both metallic wires and metal-coated fibers can be used as interconnects in fabrics to electrically connect rigid conventional electronic devices, such as electronic modules,^40^ or as electrodes to fabricate fiber-based devices,^41^ such as solar cells, lithium-ion batteries, supercapacitors, sensors, and light-emitting devices. The metallic wires, fibers, or fiber-based devices are integrated into textiles using conventional textile manufacturing techniques, such as weaving, knitting and embroidery, while fully retaining textile structures and easily forming patterns. However, breakage can occur if the sewability (strength and flexibility) of the wires or fibers cannot meet the requirements of industrial textile manufacturing machines.

**Figure 3:**
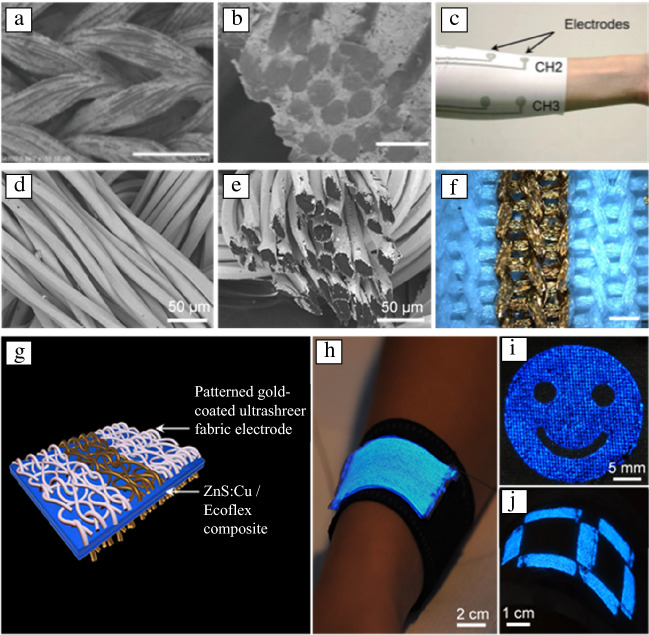
Conductive metallic textiles and textile-based electronic devices. (a) Scanning electron microscopy (SEM) image of the surface of a textile after the permeation of the specialized ink. Scale bar = 300 μm. (b) Cross-sectional SEM image of the fiber bundle after printing. Scale bar = 30 μm. (c) Photograph of the stencil-printed textile electrode. (d) SEM image of the surface of a textile after electroless deposition (ELD). (e) Cross-sectional SEM image of the fiber bundle after ELD. (f) Optical microscope image of the metal pattern on a textile. Scale bar = 400 μm. (g) Schematic of the structure of a patterned light-emitting textile. (h) Photograph of the light-emitting textiles conformal to a human arm. (i) Photograph of a light-emitting textile displaying the “smiling face” emoji. (j) Photograph of a light-emitting textile displaying the number 8; (a–c) Reprinted with permission from Reference 44. © 2017 Wiley. (d–f) Reprinted with permission from Reference 49. © 2018 Wiley. (g–j) Reprinted with permission from Reference 13. © 2020 Elsevier.

A current focus in metallic e-textiles is the development of scalable deposition methods that coat individual yarns or fibers with metal-based materials to maintain the void spaces and thus the softness and stretchability of the textile. Solution-based methods are a promising approach: solutions wick into textiles via the capillary force generated from the gaps between fibers, thereby having the potential to access each individual fiber.^42^ One solution-based approach involves formulating a conductive ink of elemental metal nanomaterials, solvent, and/or binder and then applying the metal-based ink onto textiles through printing or dip coating.^43–46^ The properties of the ink (such as viscosity and surface tension) and the properties of the textile (such as porosity and coating factor of the textiles and hydrophilicity of textile fiber materials) control the ink penetration depth, which has a significant effect on the electrical and mechanical properties of the coated textiles.^47,48^ When the ink cannot penetrate into the fabric well, it becomes localized at the surface and fills the fabric voids, stiffening the fabric and creating a conductive coating that is vulnerable to cracking. To avoid these issues, Jin et al. precisely formulated a textile-permeable viscous ink by adjusting the evaporation rate using different solvents.^44^ The specialized ink only filled the small gaps between individual fibers, leaving the large voids between fiber bundles unchanged to retain flexibility and stretchability (**Figure ****3**a–b). Another advantage of this approach is the ability to use low-cost and scalable printing techniques to create conductive patterns. Using stencil printing, the authors fabricated stretchable and conductive patterns on textiles with a sheet resistance of 0.06 Ω sq^–1^ and stretchability of 450% strain at which the resistance increases 70 times (Figure 3c).

Rather than using ink formulation to control permeation, an alternative approach uses an aqueous metal deposition solution that fully permeates into textile structures to deposit a metal coating onto the surfaces of individual textile fibers through the reduction of metal ions.^35,49–53^ This approach can be thought of as analogous to the dyeing process used in the textile industry. Both processes deposit material onto fiber surfaces while retaining the original void structure of the textile and both are compatible with low-cost printing technologies. Simple metallization solutions include metal ions or complexes that adhere to textile surfaces and are subsequently reduced to form a metallic coating by thermal reduction. For example, silver-coated textiles have been fabricated by dyeing the fabric with silver ion solution followed by thermal reduction to elemental silver,^52^ and inkjet printing an organometallic silver complex followed by thermal reduction to elemental silver to produce stretchable silver traces.^51^ Although this approach produces conductive textiles that retain the intrinsic flexible, stretchable, and breathable properties of the fabric, producing textiles with high conductivity (sheet resistance of ~ 0.1 Ω sq^–1^) often requires multiple deposition steps. Electroless deposition (ELD) is an industrial solution metallization method widely used in printed circuit board fabrication that provides better control over the metal thickness and access to a wide range of different metals. In the ELD process, metal ions in the plating solution are chemically reduced to metal by a catalyst chemisorbed on the surface of the textile. The metal film then autocatalyzes further metal deposition as a reducing agent in the plating solution is consumed, allowing control over the film thickness by adjusting the plating time.^54^ Wu et al. used ELD methods to fabricate conductive gold-coated knitted polyester textiles and nylon textiles with sheet resistances of a few Ω sq^–1^ and conductivity that persists to the elastic strain limit of the textile (200% strain).^13,49^ The aqueous solutions used in the ELD process permeate the fabric to deposit a smooth, conformal gold coating on the individual fibers (Figure 3d), with each fiber consisting of a core–shell structure (Figure 3e). This ELD method can be combined with stencil printing of a removable wax resist to provide patterned gold-coated textiles (Figure 3f).^13,49^ Gold-coated textiles prepared by ELD have been used as an integral component to fabricate e-textile devices such as stretchable light-emitting textiles (Figure 3g–j)^13,49^ and energy-storage textiles.^55^

## Conducting polymers

Conjugated polymers comprise a π-conjugated backbone and flexible side chains, which impart processability from solution and melt. Electronic charge conduction occurs via the conjugated backbone while ion transport is facilitated by oligoether side chains^56^ or polyelectrolyte counterions such as poly(styrene sulfonate) (PSS).^57^ Most conjugated polymers are obtained in their neutral form, and as such can be used as semiconductors. An intrinsic hole or electron conducting material is created by doping (i.e., a second molecule–a molecular dopant) is added that exchanges one or several electrons or a proton/hydride (H^+^/H^–^) with the conjugated polymer.^58,59^ Some polymers such as poly(3,4-ethylenedioxythiophene) (PEDOT) are instead prepared by oxidative polymerization, which yields the highly oxidized, conducting form, complexed with a counterion or polyelectrolyte such as PSS, which can be processed as an aqueous dispersion.^60^ Conjugated polymers are widely explored for the design of solution-processed thin-film electronic devices for applications ranging from organic photovoltaics to bioelectronics.

**Figure 4:**
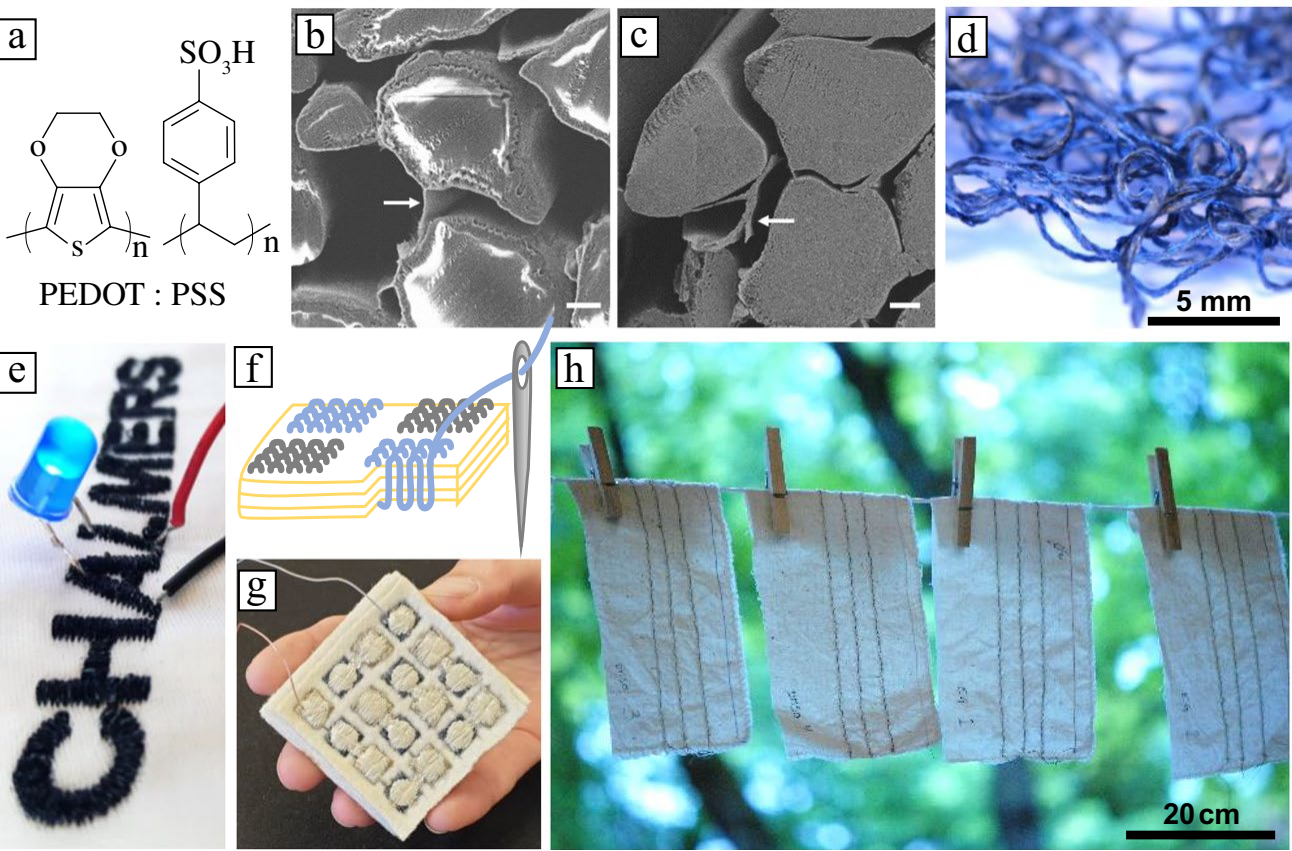
(a) Chemical formula of poly(3,4-ethylenedioxythiophene) poly(styrene sulfonate) (PEDOT:PSS). (b) SEM image of a freeze-fractured PEDOT:PSS dyed silk yarn with charging artifacts indicating penetration of the conductive coating, and (c) SEM image of freeze-fractured PEDOT:PSS dyed silk yarn (sputtered with palladium). Scale bars = 2 µm. (d) Photograph of PEDOT:PSS dyed silk yarn. (e) Electrically conducting Chalmers logo, connected to a blue light-emitting diode and a battery (not shown), machine embroidered using a PEDOT:PSS coated silk yarn. (f) Schematic of embroidery of conducting silk yarns (blue) and silver-plated polyamide thread (gray) through several layers of a wool fabric, and (g) embroidered textile thermoelectric generator. (h) PEDOT:PSS coated silk yarns stitched onto cotton swatches line drying after machine wash tests. (b–d, h) Reprinted with permission from Reference 14. © 2017 American Chemical Society. (e) Reprinted with permission from Reference 10. © 2018 Wiley. (f) Reprinted with permission from Reference 22. © 2020 Elsevier.

Conjugated polymers can be used as a dye or coating for existing textile materials, or they can be used to prepare semiconducting, conducting or electrochemically active fibers and yarns. While some polymers such as polyaniline and poly(3-hexylthiophene) (P3HT) can be both melt- and wet-spun into fibers,^61–63^ most available conjugated polymer grades have a too low molecular weight to be suitable for fiber spinning or are too brittle due to a high glass transition temperature.^64^ The rheological and mechanical properties of conjugated polymers can be adjusted through combination with a second component, which can be achieved by blending with a commodity polymer such as polyethylene or polyurethane,^65,66^ or complexation with a polyelectrolyte such as PSS,^60^ resulting in a material that can be spun into fibers. Alternatively, an already existing natural or synthetic fiber, yarn or textile can be coated, impregnated, or dyed with a conducting polymer ink such as aqueous PEDOT:PSS (**Figure ****4**a–d),^11,14,67,68^ or functionalized through *in situ* or vapor polymerization of PEDOT.^27,33,69,70^

Conducting polymer-based fibers can display a high stiffness and conductivity. PEDOT:PSS wet-spun fibers, for example, feature a Young’s modulus of up to 22 GPa and a conductivity of up to 4000 S cm^–1^.^6,71–73^ For many textile applications highly stretchable fibers with a low stiffness are required, which necessitates that the mechanical and electrical properties of the fiber are decoupled (Figure 1). Blends of a low-modulus commodity polymer such as polyurethane with a conducting polymer, or polyurethane fibers coated with a conducting polymer, can result in fibers/yarns with a high degree of stretchability and reasonably high conductivity of typically not more than about 10 S cm^–1^.^23^

A high degree of air and thermal stability as well as wash and wear resistance of conducting polymer-based fibers is essential for the realization of e-textile devices. While many conjugated polymers are stable in their neat, undoped state, a gradual decrease in electrical properties tends to occur over time. The loss of conductivity is prominent if the conjugated polymer is doped with a small-molecular dopant, such as iodine or 2,3,5,6-tetrafluoro-7,7,8,8-tetracyanoquinodimethane (F4TCNQ), which tend to sublime^74^ and therefore pose a health hazard due to their high reactivity. All-polymer materials, instead, can display a high degree stability. For example, PEDOT:Nafion stretchable fibers show no decrease in conductivity over at least 1  year.^70^ Blend fibers can be expected to display superior wear resistance since the conducting material is protected by the insulating polymer matrix. Further, highly wash and wear resistant coatings have been reported. For instance, PEDOT:PSS dyed silk yarns can withstand both machine-washing and dry cleaning,^14^ as well as the abrasive wear experienced during textile manufacturing (Chapter 2),^10^ suitable for the realization of embroidered devices such as conducting patterns (Figure 4e) or textile thermoelectric generators (Figure 4f–h).^22^

Undoped conjugated polymers can be used as the active semiconducting layer in thin-film devices such as solar cells and light-emitting diodes, which can be fabricated onto nonplanar surfaces, including monofilaments. Even though prototype devices such “solar power wires” consisting of 100-μm-thin stainless-steel wires coated with layers of conjugated materials have been demonstrated,^75^ we argue that the intricate device design and delicate nature of thin-film devices would complicate the integration in daily use textiles.

Conjugated polymers are highly versatile because they are able to conduct both ions and electrons. Therefore, conducting polymer fibers and yarns can be used for the fabrication of devices that rely on electrochemical reactions, where the conducting component is brought in contact with an electrolyte. Possible electrochemical devices include woven circuits based on organic electrochemical transistors (OECTs),^8^ fiber-based sensors,^6^ knitted or woven actuators,^26^ electrochromic textiles,^68^ and energy-storage devices such as textile-based supercapacitors.^76–78^ One advantage of electrochemical devices is the insensitivity of the operating voltage to the device dimensions, meaning that they can accommodate differences in the positioning of fibers, which occur due to the limited precision of textile manufacturing as well as deformation.^23^

The vast majority of device designs that have been explored incorporate the conjugated polymer as a semiconductor or electrochemically active material, while electrical connectors are usually fabricated with more conductive materials, especially metals. Alternatively, hybrid materials can be used such as silk or cellulose yarns with a composite coating of silver nanowires and PEDOT:PSS, which display a conductivity of up to 320 S cm^–1^ as well as a high degree of wash and wear resistance.^32,67^ We conclude that the most promising applications are likely realized by combining different classes of materials.

## Graphene, related 2D materials, and hybrids

Graphene and related 2D materials have emerged as key enabling elements for high-performance wearable electronics and smart textiles.^79^ Graphene shows unique electronic properties, including semi-metallic behavior and superb room-temperature hole mobility of up to 2.5 × 10^5^ cm^2^ V^–1^ s^–1^.^80^ Other related 2D materials, including hexagonal-boron nitride (h-BN),^81^ transition-metal dichalcogenides (TMDs),^82^ and MXenes,^83^ display insulating, semiconducting and metallic behavior and have recently reshaped the landscape of device architectures.^84^

Crucially, 2D materials can be mass-produced from solution offering a new platform for manufacturing inks and dispersions enabling smart textile devices.^85^ Ultrasonication, shear forces or an electrochemical potential can exfoliate a bulk layered material into a distribution of single and few-layer flakes. Liquid phase exfoliation (LPE), is a common, scalable technique for producing solution-processable 2D materials with relatively low basal plane defects.^86^ Alternatively, graphene oxide can be produced via the modified Hummers’ method in scalable, surfactant free, high concentration water-based dispersion.^87^ However, additional thermal or chemical reduction steps are required to reduce basal plane defects and restore some electrical conductivity in the reduced graphene oxide.^88^ Furthermore, MXenes are produced by the selective etching of Group 13–16 elements from the parent MAX phase.^89^ Although their initial production was hampered by the hazardous etching process,^83^ more recent, greener techniques have opened a path toward scalable production of MXenes for integration into smart textile devices.^90^

Functional textiles dip coated with inks of 2D materials (whereby textiles are immersed in dispersions of the 2D material followed by drying) have resulted in textile strain and electrocardiogram (ECG) sensors.^4,91^ Nylon textiles coated with reduced graphene oxide with an electrical conductivity of about 5 S cm^–1^ could be used as gel-free electrodes for recording of ECGs, which achieved 97% cross-correlation when compared to conventional silver/silver chloride (Ag/AgCl) reference electrodes.^4^ Similarly, vacuum filtration may also be used to deposit active materials on the textiles to produce strain sensors^92^ or electromagnetic shielding.^93^

**Figure 5:**
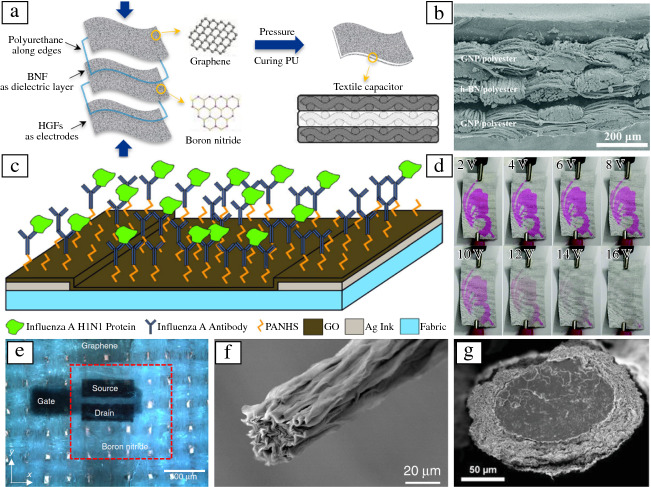
(a, b) Layered dip coating technique to produce a parallel-plate capacitor on polyester, using graphene nanoplatelets as the electrode material, and h-BN as the dielectric material. (c) Screen-printed impedimetric biosensor on cotton. (d) Electro-thermochromic butterfly patterns as a function of the applied voltage across the graphene-nanoplatelet fabric. (e) High-resolution graphene field-effect transistor in an inverted-staggered configuration, fabricated by inkjet printing on polyurethane-planarized cotton, with poly(3,4-ethylenedioxythiophene) poly(styrene sulfonate) acting as the gate material and h-BN as the dielectric. (f) SEM image of an all MXene Ti_3_C_2_T_X_ fiber. (g) SEM image of the cross-section of a MXene/polyurethane composite outer sheath surrounding the inner polyurethane core. (a, b) Reprinted with permission from Reference 95. © 2019 Royal Society of Chemistry. (c) Reprinted with permission from Reference 100. © 2018 Electrochemical Society. (d) Reprinted with permission from Reference 12. © 2020 Royal Society of Chemistry. (e) Reprinted with permission from Reference 102. © 2017 Springer Nature. (f) Reprinted with permission from Reference 21. © 2020 Springer Nature. (g) Reprinted with permission from Reference 108. © 2020 Wiley.

However, graphene oxide reduction on textiles is limited by the maximum heating threshold of the textile material used while the most efficient chemical reducing agents may pose health hazards,^94^ leaving pristine graphene a preferred option. Dip coating of polyester textiles in pristine graphene and h-BN inks (synthesized via LPE), enabled conducting (sheet resistance ≈ 2 kΩ sq^–1^) and dielectric textiles (relative permittivity ≈ 2.4), which could be used to construct solid-state capacitors based on 2D material all-textile heterostructures (**Figure ****5**a–b).^95^ The textile capacitor coupled to a graphene-polyester textile resistor demonstrated an all-textile radio-frequency (RF) low-pass filter operating at 15 kHz.^95^ Similarly, a textile triboelectric nanogenerator (TENG) can be optimized to harvest mechanical energy from the involuntary motion of skin against fabric during everyday mobility.^96^ The TENG was constructed by dipping a knitted poly(ethylene terephthalate) (PET) textile into dispersions of LPE black phosphorus followed by dip or spray coating of hydrophobic cellulose oleoyl ester nanoparticles. The resulting fabric was then combined with a fabric electrode (PET dip coated into a composite of silver and polydimethylsiloxane, PDMS), to produce an all-textile TENG, which could be used to power a digital watch.

Printing of 2D materials allows to realize complex device architectures on textiles.^85^ Screen printing, a popular technique in the textile and garment industry, has been used to pattern geometries enabling antennas, supercapacitors, and sensing devices on textile substrates.^97–99^ The excellent sensitivity of graphene to external elements is key to enabling of textile biosensors. Screen-printed graphene oxide electrodes on cotton textile functionalized with influenza-A antibodies resulted in a biosensor with a detection limit of only 10 ng mL^–1^ (Figure 5c).^100^ More recently, electrochromic textile displays were achieved by screen-printing a pristine graphene ink on polyurethane coated cotton, with a second thermochromic polyurethane layer screen printed to the back of the conductive fabric.^12^ Applying a bias of 12 V to the conductive fabric raised the temperature to 43°C, causing a reversible color change of the thermochromic pattern defined on the fabric surface (Figure 5d).

Inkjet printing has also simplified the patterning and deposition of 2D material heterojunctions on textiles.^101^ All-inkjet printed graphene field-effect transistors (FETs) were achieved on textiles using h-BN as the dielectric, and PEDOT:PSS as the electrodes (Figure 5e).^102^ The device performance was improved by adding a 12-µm-thin polyurethane coating as a planarization layer to the textile, which drastically reduced the surface roughness. These graphene FETs displayed a hole mobility of 91 cm^2^ V^–1^ s^–1^, which is several orders of magnitude higher than values reported for polymer devices on textiles.^102,103^ Importantly, the devices remained operational after at least 20 washes. The authors also demonstrated complementary inverters, and logic gates with graphene/h-BN FETs, which proves the viability of these devices for printed and textile integrated circuits.^102^

An alternative approach to depositing 2D materials onto textiles is the direct integration of the 2D materials in the textile fibers, such as by growing graphene on metal template meshes^104^ or fiber spinning.^23,105^ In recent years, improvements in strength, stiffness, flexibility, and toughness have allowed pure 2D material electronic fibers to be knitted into electronic textiles. For example, graphene oxide fibers have been created through dry-jet wet-spinning by careful control of the coagulation conditions, resulting in strong fibers with a Young’s modulus of 8 GPa. However, the electrical properties were not reported in this work.^106^ Similarly, meter-long pure MXene Ti_3_C_2_T_X_ fibers (Figure 5f) have been realized with a Young's modulus of 30 GPa and an electrical conductivity of σ ≈ 7700 S cm^–1^, higher than their MXene/graphene (σ ≈ 290 S cm^–1^) or MXene/polymer (σ ≈ 1490 S cm^–1^) counterparts.^21^

For many device designs 2D material composite structures are preferred, which can display enhanced functionalities and superior mechanical properties suitable for integration with textiles. A graphene silver hybrid fiber with a conductivity of up to 1.5 × 10^4^ S cm^–1^ was employed as the electrode material for planar and fiber-based transistors.^107^ Knitted fiber-based strain sensors using MXene/polyurethane coaxial fibers (Figure 4g) could be used to sense strains as large as 200% with a gauge factor of about 8 (more than 1 × 10^4^ for single fibers).^108^

## Outlook

E-textile materials and devices can be realized with a wide range of metals, conducting polymers, carbon allotropes and 2D materials, all of which offer unique advantages but are, on their own, also associated with unique challenges. Conducting polymers are the least conducting type of material but are electrochemically active, which offers the possibility to interface e-textiles with biological systems. Carbon allotropes and in particular metals are more conducting but are associated with a high stiffness, which is in many cases undesirable for the design of pliable and stretchable textiles.

In combination with each other, and/or synthetic polymers or natural materials such as silk or cellulose, unique types of materials can be created that are optimal for textile production, display a high degree of wash and wear resistance, and feature the pliability and stretchability that characterize traditional textile materials. Examples that have been discussed include silk yarns with a conducting polymer/metal coating, stretchable polymer nanocomposite filaments, as well as metal-coated yarns and fabrics. These different types of conducting, semiconducting or electrochemically active fibers, yarns and fabrics display promising form and function, ideal for the realization of a wide range of e-textile devices.

While each component can be reused, the combination of different materials poses a significant challenge in terms of recycling at the end of their lifetime. We argue that energy recovery is not a valid approach. Instead, future research efforts should concentrate on the design of hybrid materials that can be easily separated into their individual components, i.e. metals, polymers and 2D materials, without compromising their electrical, electronic and electrochemical properties. In order to facilitate truly sustainable e-textile materials, it would be advantageous to limit the number of different materials that are combined. Sustainability should be a key requirement at each stage of the life cycle from e-textile production, to usage, waste collection and recycling.

Even though not every challenge related to the sustainable use of e-textiles can be addressed through materials science, we anticipate that technological solutions will play a major role. For instance, while many e-textile materials have been extensively tested on the laboratory scale, including wash and wear resistance tests, it is not always clear how gradual degradation will affect the recyclability of different classes of materials. Hence, dedicated accelerated aging studies must be carried out. Further, the use of potentially toxic compounds and solvents should be avoided, to protect workers at the production and recycling stages as well as, of course, the user who will be in frequent contact with the e-textile. Questionable compounds include highly reactive oxidizing and reducing agents that are used to dope conjugated polymers, some carbon allotropes such as, arguably, carbon nanotubes, as well as some metal nanoparticles. More work is needed to identify which materials are safe to use in an e-textile context.
